# A Prospective Study Comparing Three-Dimensional Rectal Water Contrast Transvaginal Ultrasonography and Computed Tomographic Colonography in the Diagnosis of Rectosigmoid Endometriosis

**DOI:** 10.3390/diagnostics10040252

**Published:** 2020-04-24

**Authors:** Fabio Barra, Ennio Biscaldi, Carolina Scala, Antonio Simone Laganà, Valerio Gaetano Vellone, Cesare Stabilini, Fabio Ghezzi, Simone Ferrero

**Affiliations:** 1 Academic Unit of Obstetrics and Gynecology, IRCCS Ospedale Policlinico San Martino, 16132 Genoa, Italy; fabio.barra@icloud.com; 2 Department of Neurosciences, Rehabilitation, Ophthalmology, Genetics, Maternal and Child Health (DiNOGMI), University of Genoa, 16132 Genoa, Italy; 3 Department of Radiology, Galliera Hospital, 16142, Genoa, Italy; ennio.biscaldi@gmail.com; 4 Unit of Obstetrics and Gynecology, Gaslini Institute, 16147 Genova, Italy; carolinascala@icloud.com; 5 Department of Obstetrics and Gynecology, “Filippo Del Ponte” Hospital, University of Insubria, 21100 Varese, Italy; antoniosimone.lagana@uninsubria.it (A.S.L.); fabio.ghezzi@uninsubria.it (F.G.); 6 Department of Surgical and Diagnostic Sciences, IRCCS Ospedale Policlinico San Martino, 16132 Genoa, Italy; vgvellone@gmail.com (V.G.V.); cesare.stabilini@unige.it (C.S.)

**Keywords:** rectosigmoid endometriosis, three-dimensional rectal water contrast transvaginal ultrasonography, computed colonography, bowel stenosis, bowel endometriosis, intestinal segmental resection

## Abstract

(1) Objectives: In patients with symptoms suggestive of rectosigmoid endometriosis, imaging techniques are required to confirm the presence and establish the extent of the disease. The objective of the current study was to compare the performance of three-dimensional rectal water contrast transvaginal ultrasonography (3D-RWC-TVS) and computed tomographic colonography (CTC) in predicting the presence and characteristics of rectosigmoid endometriosis. (2) Methods: This prospective study included patients with suspicion of rectosigmoid endometriosis who underwent both 3D-RWC-TVS and CTC and subsequently were surgically treated. The findings of imaging techniques were compared with surgical and histological results. (3) Results: Out of 68 women included in the study, 37 (48.9; 95% C.I. 38.2–59.7%) had rectosigmoid nodules and underwent bowel surgery. There was no significant difference in the accuracy of 3D-RWC-TVS and CTC in diagnosing the presence of rectosigmoid endometriotic nodules (*p* = 0.118), although CTC was more precise in diagnosing endometriosis located in the sigmoid (*p* = 0.016). 3D-RWC-TVS and CTC had similar precision in estimating the largest diameter of the main endometriotic nodule (*p* = 0.099) and, in patients undergoing segmental resection, the degree of the stenosis of the bowel lumen (*p* = 0.293). CTC was more accurate in estimating the distance between the lower margin of the intestinal nodule and the anal verge (*p* = 0.030) but was less tolerated than 3D-RWC-TVS (*p* < 0.001). (4) Conclusion: This was the first study comparing the performance of 3D-RWC-TVS and CTC in the diagnosis of rectosigmoid endometriosis. Both techniques allowed for the evaluation of the profile of the bowel lumen in a pseudoendoscopic fashion and had a similar performance for the diagnosis of rectosigmoid endometriosis, although CTC was more accurate in diagnosing and characterizing sigmoid nodules.

## 1. Introduction

Rectosigmoid endometriosis is one of the most severe forms of endometriosis, and it is defined by the presence of endometriotic glands and stroma infiltrating at least the muscularis propria of the rectosigmoid colon. Besides pain symptoms, this disease may cause several intestinal complaints that often worsen during the menstrual cycle (such as painful bowel movements, abdominal bloating, cyclical diarrhea or constipation, passage of mucus with the stools and rectal bleeding) [1]. An accurate preoperative diagnostic workup of rectosigmoid endometriosis is necessary to provide the patient with informed consent on the benefits and risks of the potential treatments (hormonal therapies or surgical approach) and to obtain adequate informed consent in case of surgery [2,3]. Furthermore, knowing the presence of bowel endometriosis preoperatively allows planning surgery with an appropriate multidisciplinary team, including the colorectal surgeon. Finally, the features of intestinal nodules may be relevant for some surgeons to choose the technique used to excise rectosigmoid nodules (shaving, discoid excision, or segmental bowel resection) [4,5].

Several ultrasonographic and radiological techniques (such as transvaginal ultrasonography (TVS), magnetic resonance imaging (MR), and rectal endoscopic ultrasonography) have been used for diagnosing rectosigmoid endometriosis [6]. In most of these techniques, the intestinal lumen is not distended, and, therefore, it is not possible to reliably estimate the degrees of stenosis caused by rectosigmoid nodules. This parameter is relevant for patients undergoing surgery, as it may affect the surgical technique. In addition, the degree of stenosis of the intestinal lumen is important for patients undergoing hormonal therapies in order to predict the risk of stenosis and occlusive symptoms during treatment. Finally, this parameter is relevant for patients desiring to conceive to minimize the risk of intestinal occlusion during pregnancy [7,8,9,10,11].

TVS is the first-line imaging technique in patients with suspicion of rectosigmoid endometriosis and, when performed by expert ultrasonographers in referral centers specializing in the diagnosis of endometriosis, it may provide most of the information useful to the clinicians [6]. Over the last ten years, several ultrasonographic techniques based on the distention of the vagina and/or rectosigmoid with saline solution and/or ultrasonographic gel have been proposed with the aim of improving the diagnosis of deep infiltrating endometriosis [12]. Among the others, rectal water contrast transvaginal ultrasonography (RWC-TVS) has been employed in this setting, demonstrating a high accuracy in ruling out the presence of rectosigmoid endometriosis [13,14,15].

Three-dimensional (3D) reconstructions convert standard 2D grayscale acquisitions into a volumetric dataset. 3D ultrasound has been employed for the evaluation of many gynecological diseases, including uterine shape abnormalities (e.g., Mullerian duct abnormalities), uterine intracavitary pathology (submucous uterine fibroids or endometrial polyps) [16]. The use of 3D-TVS has also been proposed for the diagnosis of deep endometriosis [17,18,19].

In general, radiological techniques can be used to establish the presence and extent of rectosigmoid endometriosis, particularly when there is suspicion of disease despite negative ultrasonographic findings and/or of the presence of multifocal disease with sigmoid or upper intestinal nodules. Moreover, in order to plan surgery, nowadays the role of radiologic imaging still remains relevant and many patients with clinical suspicion of rectosigmoid endometriosis are routinely referred to radiologists for the diagnosis of intestinal endometriosis [20,21].

Computed colonography (CTC) is used worldwide for the screening of colorectal cancer. Over the last ten years, several studies showed that CTC has high diagnostic performance in the diagnosis of rectosigmoid endometriosis [22,23,24,25,26]; this exam is as accurate as TVS in diagnosing rectosigmoid endometriosis and has the advantage of investigating the whole colon [27]. Image post-processing is performed using workstations suitable for 3D data management and reconstruction. The evaluation of CTC also includes the anatomic reconstruction by 3D images; in case of large bowel evaluation, 3D review typically refers to an optical colonoscopy-like endoluminal fly-through (FT) of a 3D reconstructed colon.

At the best of our knowledge, no previous study has compared the diagnostic performance of 3D-rectal water contrast transvaginal ultrasonography (3D-RWC-TVS) and CTC in the diagnosis of rectosigmoid endometriosis. As both techniques are based on the distention of the rectosigmoid and allow the evaluation of the profile of the bowel lumen in a pseudoendoscopic fashion, the objective of the current study was to compare the diagnostic performance of 3D-RWC-TVS and CTC in predicting the presence and characteristics of rectosigmoid endometriosis.

## 2. Materials and Methods

The primary objective of the study was to compare the performance of 3D-RWC-TVS and CTC in the diagnosis of rectosigmoid endometriosis. The secondary objectives were to compare the precision of the two techniques in estimating the length (mid-sagittal diameter) of the intestinal nodules, the presence of multifocal disease (presence of one or more lesions affecting the sigmoid colon that are associated with a colorectal primary lesion) and the distance between the lower margin of the nodules and the anal verge.

This was a prospective study performed between March 2017 and September 2019. Subjects of the study were recruited among patients referred to our institution because of pain and intestinal symptoms suggestive of rectosigmoid endometriosis. Some patients had histological diagnosis of pelvic endometriosis during previous surgery. Previous surgical diagnosis of intestinal endometriosis, previous radiological diagnosis of intestinal endometriosis (based on MR or double-contrast barium enema), history of colorectal surgery (except appendectomy), contraindications to bowel preparation or CTC (such as non-compliant patients), previous bilateral ovariectomy, or psychiatric disorders were exclusion criteria for this study.

Study patients underwent 3D-RWC-TVS performed by an ultrasonographer highly skilled in the diagnosis of deep endometriosis. CTC was done within the following three months by a radiologist expert in the diagnosis of deep endometriosis, blinded to the results of the previous ultrasonographic exam.

As described in the consensus opinion from the International Deep Endometriosis Analysis (IDEA) group, intestinal nodules located below the level of the insertion of the uterosacral ligaments on the cervix were defined as “anterior lower rectal nodules”, those above this level were defined as “anterior upper rectal nodules”, those at the level of the uterine fundus were defined as “rectosigmoid junction nodules” and those above the level of the uterine fundus were defined as “anterior sigmoid nodules” [6]. Bowel stenosis was defined as a reduction in lumen, by measuring the smallest stricture diameter and comparing it with the closest healthy bowel lumen diameter.

Patients underwent surgery within six months from the performance of 3D-RWC-TVS. The results of CTC and 3D-RWC-TVS were compared with surgical and histological findings.

The local ethics committee approved the study protocol (CE2439PRNO161219-24/2020). Patients participating in the study provided written informed consent. This study was registered in Clinicaltrial.gov (NCT04295343).

### 2.1. Three-Dimensional Rectal Water Contrast Transvaginal Sonography

A sonographer (S.F.) with extensive experience in the diagnosis of intestinal endometriosis (>500 exams every year) performed all the exams. 3D-RWC-TVS was performed by Voluson E6 and E10 machines equipped with transvaginal transducers (GE Healthcare Ultrasound, Milwaukee, WI, USA). Patients received a rectal enema (133 mL of monobasic sodium phosphate anhydrous; Clisma Lax; Sofar, Milan, Italy) a few hours before TVS. A total of 300–400 mL of saline solution was employed to distend the rectosigmoid under ultrasonographic control, by using a catheter connected to a 100 mL syringe introduced in the anus [14,28].

**Figure 1:**
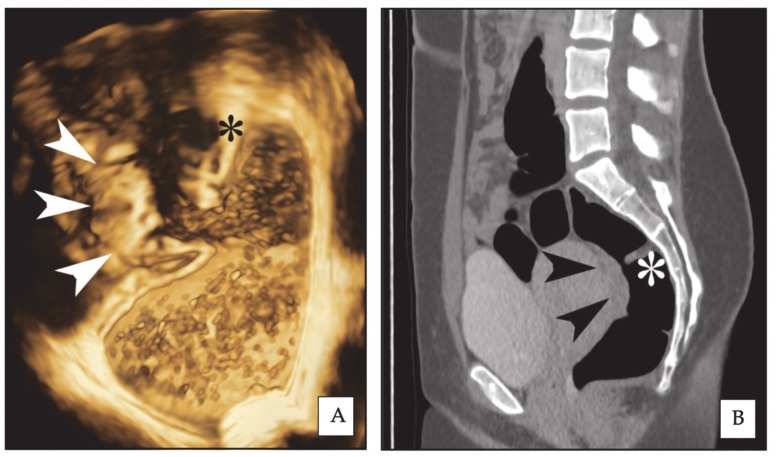
Same rectal endometriotic nodule (arrowheads) is shown in three-dimensional rectal water contrast transvaginal ultrasonography (3D-RWC-TVS) (**A**) and computed tomographic colonography (CTC) (**B**, sagittal plane). The asterisk indicates the same rectal Houston’s valve. The nodule has largest diameter of 2.6 cm.

During the ultrasonographic scan, acquisitions of images by 3D rendering were made in the sagittal and coronal planes. Two specific quality enhancement tools (GE Healthcare Ultrasound, Milwaukee, WI, USA) were applied during 3D rendering: advanced Speckle Reduction Imaging (SRI), which helps heighten the visibility of lesions with high-definition contrast resolution and CrossXBeamCRI^TM^, which improves the enhancement of tissue and border differentiation. On the 3D rendering, rectosigmoid lesions typically appear as spiculated lesions with a retracting line all around the nodule (Figure 1) [18]. Multiple acquisitions were performed to characterize the endometriotic nodules, in particular, measuring their largest diameter and their distance from the anal verge. When the volume acquisition was completed, the data file was sent via Digital Imaging and Communication in Medicine (DICOM) to a personal computer and stored in order to be analysed by the use of an appropriate software (4Dview 5.0; GE Healthcare Ultrasound, Milwaukee, WI, USA). All the acquisitions were examined by another sonographer who has performed over 1000 analysis of 3D imaging related to deep endometriosis in his life (F.B.). This sonographer was blinded to the results of the 2D-RWC-TVS. For estimating the stenosis, at least three measurements of the diameter of rectosigmoid lumen were performed above and below the nodule (mean of all measurements) in a healthy bowel; close to the nodule surface, at least one measurement every 5 mm was performed (mean of the three lower measurements).

### 2.2. Computed Colonography

Patients underwent a low residue diet and a liquid diet on the three days before and in the 24 h before CTC, respectively. On the afternoon and the evening of the day before the exam, the patients had an intestinal preparation, which consisted in sodium picosulfate (10.0 mg), light magnesium oxide (3.5 g) and anhydrous citric acid (10.97 g) (CitraFleet, CasenRecordati SL, Zaragoza, Spain). Patients were asked to drink a dose of diatrizoatemeglumine and diatrizoate sodium solution (Gastrografin; Schering, Berlin, Germany) diluted 1:1 with tap water at 6 p.m. on the day before the exam.

A radiologist (E.B.) with extensive experience in the diagnosis of intestinal endometriosis (>500 exams every year) performed all the exams.

The scans were performed by using a 64-section multidetector CT scanner (LightSpeed, GE Healthcare, Milwaukee, WI, USA) according to a standardized protocol [27]. A 12F Foley catheter was introduced into the distal rectum before the scan on the CT bed, and 2.5–3 L of room air were introduced in the colon, calculating the same number of manual pomp inflations in all the distensions. The patients were scanned in the supine and prone positions. No intravenous injection of iodinated contrast medium was employed. The abdomen was scanned as in conventional CTC performed for rectal cancer screening.

The DLP (dose length product) of CTC depended on the length of the abdominal surface of the women that is related to her height. The estimated radiation dose was in the range of 6 mSv. The adaptive statistical iterative reconstruction (ASIR; GE Healthcare) was used to decrease the x-ray dose without a significant loss of image quality.

A weight-based automated tube-current modulation technique was employed with a tube current range of 130–150 mA for patients weighing less than 70 kg in order to decrease the effective radiation dose (abdominal volume acquisition wCTDi = 6–7 mSv). Collimation was 1.25 mm with a helical pitch of 1.375; the reconstruction interval (overlap) was 1 mm. The raw data were transferred to a workstation having a dedicated CTC software package (General Electric ADW 4.2.4, General Electric Medical System, Milwaukee, WT, USA).

Post-processing image editing was performed, being the scans evaluated by various reconstructions: the 3D endo-luminal FT and virtual dissection reconstructions allowed for the visualization of the lumen of the rectum, sigmoid, and the other parts of the colon. 3D images were always used in case of problem solving; the diagnosis was performed on the basis of axial images and multiplanar reconstructions (MPRs).

**Figure 2:**
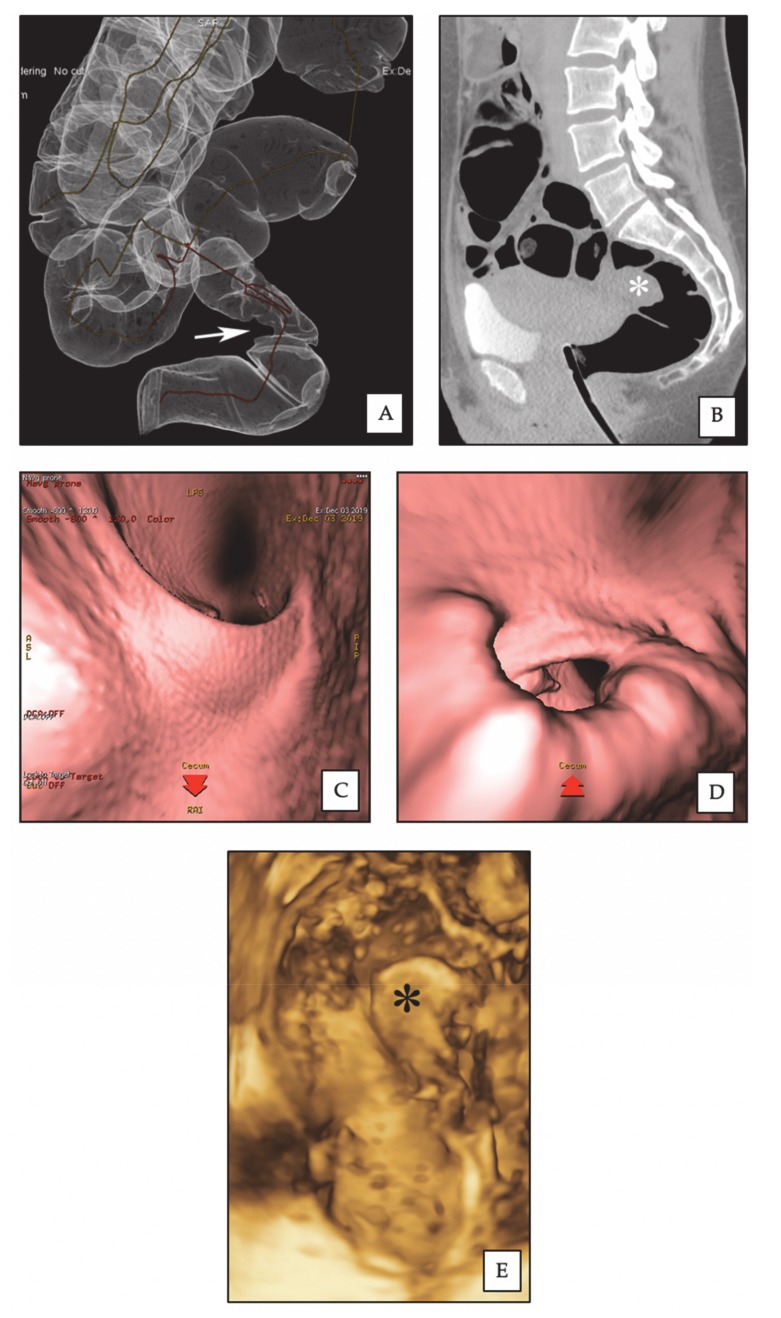
Rectal endometriotic nodule. (**A**) CTC: 3D reconstruction of dilated colon, showing rectal stenosis (arrow) by an endometriotic nodule. (**B**) CTC: sagittal 2D image, the rectal nodule (asterisk) causes stenosis of the intestinal lumen. (**C**) CTC: pseudoendoscopic view and 3D endoluminal fly-through reconstruction, showing normal rectal lumen. (**D**) CTC: pseudoendoscopic view and 3D endoluminal fly-through reconstruction, showing rectal stenosis by the endometriotic nodule. (**E**) 3D-RWC-TVS showing the rectal nodule (asterisk). The nodule has a largest diameter of 2.8 cm; the distance between the lower margin of the nodule and the anal verge is 10 cm.

Rectosigmoid endometriotic nodules appear on CTC as strictures usually involving a variable part of the circumference of the bowel wall (Figure 1); the site of these findings is constant on both the supine and prone scans and stenosis may be highlighted by the 3D endo-luminal FT reconstructions (Figure 2). The MPRs sometimes allow for the detection of a transmural involvement of the endometriotic nodule [27].

For estimating the stenosis, at least one measurement of the diameter of rectosigmoid lumen was performed every 10 mm in the 50 mm above and below the nodule (mean of all measurements) in healthy bowel; on the nodule surface, at least one measurement every 5 mm was performed (mean of the three lower measurements).

### 2.3. Tolerability of Radiological Exams

After each exam, the intensity of the pain perceived was rated by each patient by using a 100-mm visual analogue scale (VAS) scale. Furthermore, patients were asked to qualitatively rate the discomfort perceived during the exam by a 5-point Likert scale (very tolerable, tolerable, neutral, painful, very painful).

### 2.4. Surgical Procedures

The surgeons were aware of the findings of the 3D-RWC-TVS and CTC. A team of gynecological and colorectal surgeons performed all the procedures by laparoscopy. The rectosigmoid nodules were excised by shaving (excision of the nodule with cold scissor or monopolar hook without entering the intestinal lumen), discoid excision (removal of the nodule with opening of the intestinal wall and suture of the bowel) or by segmental bowel resection. The retrieval of surgical specimens was done through laparoscopic accesses by using an endo-bag (the laparoscopic accesses may have been enlarged in case of large specimen). The morcellation of surgical specimens was always avoided. During laparoscopy, the distance between the main rectosigmoid endometriotic nodule and the anal verge was estimated by introducing a 20-F soft rectal catheter within the rectum up to the level of the intestinal lesion. In case of laparoscopic shaving, a rectal probe was employed for exposing better the endometriotic lesion. In patients who underwent segmental bowel resection, the stenosis of bowel lumen caused by endometriosis was evaluated.

### 2.5. Statistical Analysis

Accuracy, sensitivity, specificity, positive predictive value (PPV) and negative predictive value (NPV) were evaluated for 3D-RWC-TVS and CTC. The diagnostic value of each technique was also assessed by calculating the positive likelihood ratio and negative likelihood ratio. Efficacy parameters were calculated with 95% confidence intervals (CIs). The accuracy of 3D-RWC-TVS and CTC in the diagnosis of intestinal endometriosis was compared by using the McNemar’s test with the Yates continuity correction. The precision of the measurements of nodule size and distance from the anal verge by imaging techniques was calculated by subtracting the size of the nodule, as measured by the imaging techniques, from the size of the nodule, as measured at histology. The limits of agreement were calculated as mean difference ± 2 standard deviations of the difference. Correlations between nominal categories were estimated by phi coefficient. The normality of distribution of continuous variables was evaluated by the Kolmogorov–Smirnov normality test. The pain intensity experienced by the patients during both exams was evaluated by the nonparametric Mann–Whitney test. The data were analyzed using the SPSS software version 24.0 (SPSS Science, Chicago, IL, USA). *p* values < 0.05 was considered statistically significant.

## 3. Results

### 3.1. Characteristics of the Study Population

Table 1 shows the demographic characteristics and Supplementary Table S1 presents the main symptoms complained by the study population. Out of the 68 women included in the study, 37 (48.9; 95% C.I. 38.2–59.7%) had rectosigmoid nodules and underwent bowel surgery. The main nodules were located on the sigmoid in 16 (43.2%) patients, on the rectosigmoid junction in four (10.8%) patients, on the upper rectum in 10 (27.0%) patients and on the lower rectum in seven patients (18.9%). Twelve patients (32.4%) underwent shaving of the colorectal nodules; nine (24.3%) underwent discoid excision; 16 patients (43.2%) underwent segmental colorectal resection; in this last group of patients, the mean (± SD) length of the resected bowel specimen was 11.5 ± 1.9 cm. Concerning the depth of infiltration of endometriosis in the intestinal wall, at histology, the disease infiltrated only the muscularis mucosae in 33 patients (75.0%), the submucosa in eight patients (18.2%) and the mucosa in three patients (6.8%). Seven patients (15.9%) had multifocal disease.

### 3.2. Diagnostic Performance of 3D-RTW-TVS and CTC

3D-RTW-TVS and CTC detected 26 (70.3%) and 35 (94.6%) rectosigmoid endometriotic nodules out of 37 confirmed during surgery (Figure 2 and Table 2). There was no significant difference in the accuracy of 3D-RWC-TVS and CTC in diagnosing the presence of rectosigmoid endometriotic nodules (*p* = 0.118). However, a subgroup analysis demonstrated that CTC was more precise than 3D-RWC-TVS in diagnosing endometriosis located in the sigmoid (*p* = 0.016). In fact, 3D-RWC-TVS did not identify the presence of eight sigmoid nodules, whereas CTC did not identify only one sigmoid endometriotic nodule. The presence of an endometrioma with diameter >4 cm was positively statistically correlated to the lack of identification of sigmoid endometriotic nodules (phi coefficient 0.516; *p* = 0.039) during 3D-RWC-TVS, but not during CTC (phi coefficient 0.333; *p* = 0.182).

The mean (±SD) largest diameter of the main endometriotic nodule at histology was 22.3 (±8.7) mm. 3D-RWC-TVS and CTC estimated the largest diameter of the main endometriotic nodules (*p* = 0.099) similarly, independent of their location. The mean difference was −3.2 (±7.4) mm (95% CI, −6.0 to −0.3 mm; limits of agreement, −17.3 to 11.1 mm) at 3D-RWC-TVS and −1.0 (±2.7) mm (95% CI, −2.1 to 0.1 mm; limits of agreement, −6.4 to 4.5 mm) at CTC when compared with histology (Table 3).

At surgery, the mean (±SD) distance between the more distal rectosigmoid nodule and the anal verge was 142.7 (±45.3) mm. CTC was more accurate than 3D-RWC-TVS in estimating the distance between the lower margin of the intestinal nodule and the anal verge (*p* = 0.030). The mean difference was −16.5 (±30.1) mm (95% CI, −28.7 to −4.2 mm; limits of agreement, −75.7 to 43.4) for 3D-RWC-TVS and 3.3 (±2.0) mm (95% CI, −10.1 to 16.7 mm; limits of agreement, −57.3 to 62.1) for CTC when compared with surgery (Table 4).

**Figure 3:**
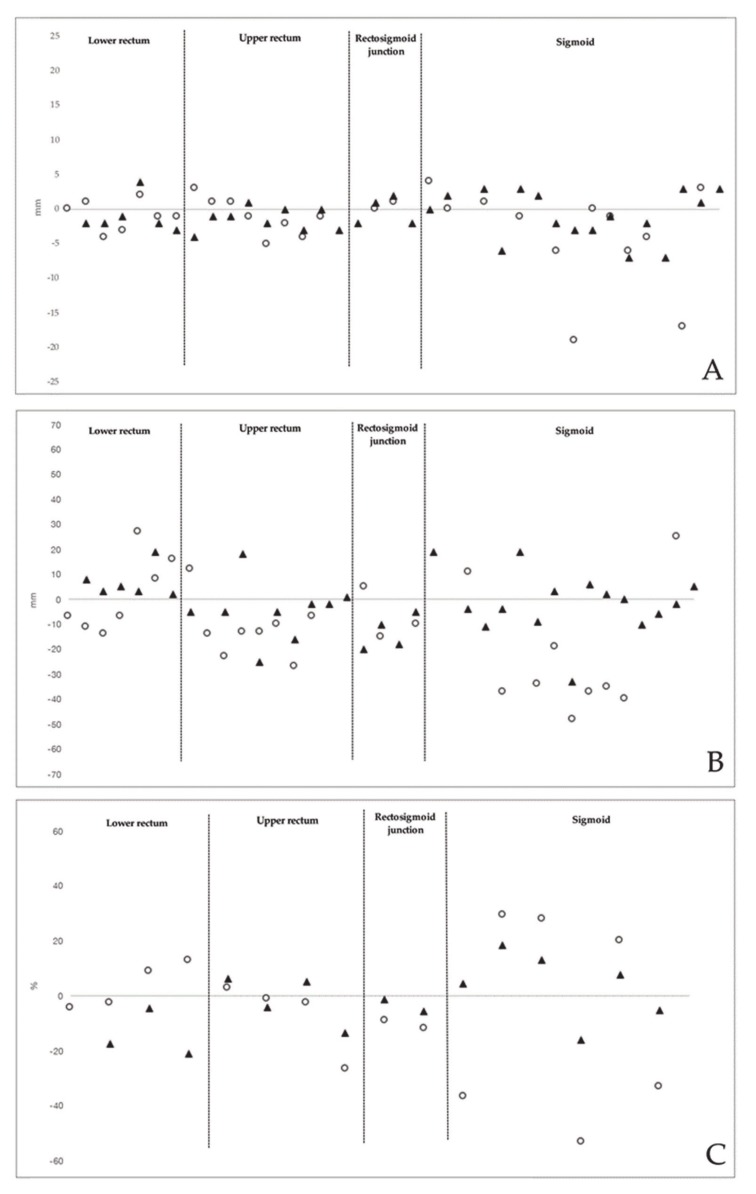
(**A**) Difference (mm) between imaging methods and surgery in estimating the largest diameter of endometriotic rectosigmoid nodules; (**B**) difference (mm) between imaging methods and surgery in estimating the distance from the lowest endometriotic rectosigmoid nodule and the anal verge; (**C**) Difference (%) between imaging methods and surgery in estimating the stenosis of bowel lumen due to the endometriotic nodules (calculated in patients undergoing colorectal segmental resection). White circles: 3D-RWC-TVS; black triangles: CTC

In patients undergoing colorectal segmental resection, at pathological examination, the degree of the stenosis of the bowel lumen was 65.1 (±21.4) %. CTC and 3D-RWC-TVS similarly estimated the degree of the stenosis of the bowel lumen (*p* = 0.293), although 3D-RWC-TVS was less accurate than CTC in determining this parameter in endometriotic nodules located in the sigmoid (*p* = 0.005). The mean difference was 17.3 (±13.8) % for 3D-RWC-TVS and 13.8 (±10.0) % for CTC in comparison to surgery (Figure 3).

CTC only identified all the cases (6/6) of multifocal disease; in one patient, 3D-RWC-TVS did not diagnose the presence of multifocal disease that was diagnosed at surgery (1/6; 16.6%). There was no significant difference in the performance of 3D-RWC-TVS and CTC in diagnosing multifocal disease (*p* = 1.000) (Supplementary Table S2).

The mean (±SD) intensity of pain experienced during CTC was higher than that perceived during RWC-TVS (VAS, 29 ± 57 vs. 15 ± 05 mm; *p* < 0.001). A higher proportion of patients complained of pain during CTC than RWC-TVS (the % of patients experiencing a painful/very painful exam was 7.5% vs. 44.1%; *p* < 0.001). 

## 4. Discussion

This is the first study comparing the performance of 3D-RWC-TVS and CTC in the diagnosis of rectosigmoid endometriosis. Both techniques are based on the distention of the rectosigmoid (with either saline solution or air/CO_2_) and allow for the evaluation of the profile of the bowel lumen in a pseudoendoscopic fashion. Moreover, 3D-RWC-TVS and CTC enable the acquisition of ultrasonographic volumetric data with unrestricted access to an infinite number of viewing planes; thus, further images can be obtained even after the first acquisition.

Our data demonstrate that 3D-RWC-TVS and CTC have similar accuracy in the diagnosis of rectosigmoid endometriosis (81.82% vs. 93.94%). However, CTC is more accurate than 3D-RWC-TVS in assessing the presence of sigmoid nodules. In fact, most of the nodules (8/11; 72.7%) not detected by 3D-RWC-TVS were located in the sigmoid; nevertheless, 62.5% of patients (6/10) had ovarian endometriotic cysts with a diameter >4 cm that may have hampered the detection of these upper intestinal nodules. Overall, thirteen false negatives procedures occurred in our study; however, in twelve cases (92.3%), the detection of endometriotic nodules was done by at least one of the two imaging techniques. These data may justify the combined use of both CTC and 3D-RWC-TVS for the workup of patients with suspicion of rectosigmoid endometriosis.

CTC was more precise than 3D-RWC-TVS in estimating the distance between the lower margin of the intestinal nodule and the anal verge: in particular, both techniques equally estimated this parameter in patients with lower, upper rectum, and rectosigmoid junction endometriotic nodules, but not in those with sigmoid nodules. Furthermore, these diagnostic exams were equally precise in estimating the largest diameter of the main rectosigmoid nodule and detecting the presence of multifocal disease.

According to IDEA consensus opinion [6], TVS should be considered the first-line imaging technique for investigating women with suspicion of rectosigmoid endometriosis, in particular, whenever performed by expert ultrasonographers in referral centers specializing in diagnosis of endometriosis. Recently, it has been reported that the need for segmental resection in patients with bowel-infiltrating nodules depends on the degree of muscular layer infiltration and corresponding thickness (muscularis rule) in addition to nodule length and can be accurately identified by preoperative ultrasonographic investigation [5]. Nevertheless, until now, nodule features and threshold values for the choice and timing of either conservative or radical surgical approach for rectosigmoid endometriosis have not been well defined [21]. In current practice, the choice of optimal surgical technique should not depend on only rectosigmoid nodule features, but also on patients’ symptoms, age, desire of conception and intention to undergo postoperative hormonal treatment for reducing the risk of disease recurrence. However, the preoperative estimation of the stenosis of rectosigmoid lumen may be helpful in the decision to plan surgery, particularly in infertile patients that may be at risk of bowel occlusion during ovarian stimulation and pregnancy [1]. In our study, CTC and 3D-RWC-TVS similarly estimated the degree of the stenosis of the bowel lumen in patients with rectosigmoid nodules undergoing segmental resection. However, 3D-RWC-TVS was again less accurate than CTC in assessing this parameter in endometriotic nodules located above the rectosigmoid junction.

CTC can characterize endometriotic rectosigmoid nodules and precisely assess the distance between their localization and the anal verge. Colonic distension with air provides a better estimation of digestive tract narrowness than that of any other imaging techniques and allows for an accurate investigation of the whole colon and, in particular, of the intestinal nodules located above the sigmoid colon (such as those on the transverse colon and the cecum) [27]. These nodules cannot be diagnosed by TVS because they are beyond the field of view of the transvaginal probe. In addition, they can also be difficult to detect when performing MR, even in the case of rectal enema (which allows for a less extended retrograde colorectal distension in comparison to CTC) [29]. Considering that more than one-third of cases of bowel lesions are multifocal [30], a complete assessment of the colon is essential to detect all endometriotic lesions for planning the surgical management; in fact, multiple endometriotic nodules on the digestive tract may require multiple segmental bowel resections or disc excisions. Furthermore, CTC is a quick outpatient exam characterized by high spatial resolution, and it allows for scanning the entire abdominal volume within seconds; this may rule out some macroscopic visceral findings (such as cysts, or calcifications) that may be relevant in the differential diagnosis of abdominal pain in patients of reproductive age. Nevertheless, CTC should not be considered as an alternative to TVS or MR, because these imaging techniques can offer a better assessment of deep pelvic endometriosis, ovarian endometriotic cysts, and uterine adenomyosis [31].

Previous studies investigated the performance of CTC in the diagnosis of bowel endometriosis [25,27,32,33,34,35]. In a prospective study including 92 women undergoing surgery for deep infiltrating endometriosis, Baggio et al. compared the diagnostic value of TVS, serum Ca125 and CTC [33]. Forty-nine subjects had rectosigmoid endometriosis. CTC had the highest accuracy in detecting bowel involvement with a sensitivity of 68% and a specificity of 67%. However, both TVS and CTC had lower performance than that previously reported by other authors, possibly because of the limited experience of the gynecologists and radiologists in the diagnosis of deep endometriosis. Zannoni et al. compared the performance of TVS and CTC in the diagnosis of rectosigmoid endometriosis in a prospective study including 47 patients with suspicion of rectosigmoid endometriosis [34]. The study showed that TVS has higher accuracy in the diagnosis of intestinal deep infiltrating endometriosis in the rectovaginal septum, rectum and sigma. In contrast, the two techniques had similar accuracy for the diagnosis of overall intestinal deep infiltrating endometriosis. Recently, a systematic review with meta-analysis investigated the performance of CTC by meta-regression, showing a global sensitivity of 93% (95% CI 84–100%) and a specificity of 91% (95% CI 81–100%) [36]. In a prospective study by our academic group, CTC was compared to RWC-TVS in 70 patients with suspicion of rectosigmoid endometriosis, obtaining a similar accuracy in diagnosing endometriotic nodules (sensitivity 90.0% and 94.3%; specificity 92.5% and 92,5%, respectively) [27]. The diagnostic performance of 3D-RWC-TVS reported in the current study is lower than that observed with RWC-TVS. In our experience, obtaining good quality 3D acquisition was more difficult in the sigmoid than in the rectum. Similar to the current data, CTC was previously found to be more precise than RWC-TVS in estimating the distance between the lower margin of the rectosigmoid nodule and the anal verge, but it was less tolerated than RWC-TVS [27].

In our prospective study, CTC was performed without the use of iodinated contrast medium in order to reduce the invasiveness of the exam. For this reason, an accurate estimation of the depth of infiltration of endometriosis in the intestinal wall was not feasible; otherwise, 3D-RWC-TVS reliably estimates this parameter [12].

Ultrasonographic study with 3D modalities has increasingly been employed for the evaluation of many gynecological diseases, being in recent years also investigated for the diagnosis of deep endometriosis [17,18,19,37]. Two previous studies prospectively compared the performance of 3D-RWC-TVS and MR with colorectal enema in the diagnosis of rectosigmoid endometriosis, finding similar accuracy parameters, nevertheless stating the advantage of an accurate visualization and characterization of the nodules by performing the 3D evaluation [38,39]. In particular, 3D-RWC-TVS is characterized by a wide spatial orientation by providing the observer with a range of different displays of the images in the three orthogonal planes. Moreover, all the acquisitions can be selected and rotated or scrolled through in fascinating virtual navigation and they can be easily assessed off-line. In our study, this advantage let us avoid a 3D real-time evaluation, which would have been subjected to the risk of bias due to 2D-RWC-TVS scan performed at the same consultation; thus, after being stored, the images acquired were interpreted by another expert ultrasonographer blinded to the results of conventional RWC-TVS and CTC imaging. Otherwise, a learning curve has to be considered because the sonographer acquired optimal expertise in 3D acquisitions and interpretation. Notably, the comparison of accuracy of 3D-RWC-TVS versus conventional 2D-RWC-TVS in the detection or characterization of rectosigmoid endometriotic nodules would be of interest. Until now, only a study published in the abstract form performed a preliminary comparison between the two ultrasonographic techniques. This study included 36 women of reproductive age with pain symptoms and intestinal complaints suggestive of rectosigmoid endometriosis. 3D-RWC-TVS and 2D-RWC-TVS had similar performance in the diagnosis of rectosigmoid endometriosis (*p* = 0.50). In particular, for the 3D-RWC-TVS, the sensitivity, specificity, positive and negative predictive values were 86.4%, 85.7%, 90.5% and 80.0%. The two techniques similarly estimated the volume of the largest intestinal nodule and the distance between the lower endometriotic bowel nodule and the anus [40].

In a similar way to the conventional 2D scan, 3D-RWC-TVS is better tolerated and less expensive than CTC. Our data show that patients experienced more discomfort during CTC than during 3D-RWC-TVS. This may be in primis due to the air distension of the colon employed during the radiologic exam, but also to the intestinal cleansing required prior to the exam, less tolerable than that employed for 3D-RWC-TVS. Another not negligible disadvantage related to the use of CTC is the exposure to X-rays; this limit is relevant since women with symptomatic endometriosis are in their fertile age and do not have an oncological pathology that would justify the radiation dose. Nevertheless, in current CTC protocols, the radiation dose has been decreased, and the average radiation administered to the patient is around 6–7 mSv that is lower than that usually used for a barium enema.

This study has some limitations. Indeed, the extensive experience of the radiologist and the gynecologist performing CTC and 3D-RWC-TVS, respectively, may have influenced the accuracy of these techniques in diagnosing rectosigmoid endometriosis. Moreover, we did not perform a power calculation for determining the sample size. As this study was based on a population of symptomatic patients with a high prevalence of intestinal symptoms, a high prevalence of rectosigmoid endometriosis was observed in this sample; subsequently, this issue may potentially have influenced the diagnostic performance of CTC and 3D-RWC-TVS. Nevertheless, the use of these diagnostic exams is justified only in symptomatic patients at high risk of having rectosigmoid endometriosis. The positive, but not statistically significative, trend in favor of CTC with regard to diagnostic parameters may be partly due to limited sample size of our study.

## 5. Conclusions

In conclusion, 3D-RWC-TVS and CTC can be used to screen the presence of rectosigmoid endometriosis, as they have comparable diagnostic accuracy. However, we deem that symptomatic patients in case of negative findings at 3D-RWC-TVS and patients undergoing surgery should also be investigated using other techniques, aiming to study the presence of sigmoid or upper intestinal nodules. Nevertheless, CTC should not be considered as an alternative to TVS or MR, because these imaging techniques provide a better assessment of deep pelvic endometriosis, ovarian endometriotic cysts, and uterine adenomyosis. In this perspective, CTC may be combined with 3D-RWC-TVS because of the high diagnostic performance in detecting rectosigmoid endometriosis and the ability to diagnose endometriotic nodules located above the sigmoid.

## Figures and Tables

**Table 1:** Demographic characteristics of the study population (*n* = 68).

Age (years; mean ± SD)	35.4 ± 6.0
Body mass index (kg/m^2^; mean ± SD)	24.7 ± 3.2
Race (*n*, %)	
• Caucasian	64 (94.1%)
• African	3 (4.4%)
• Asiatic	1 (1.5%)
Previous live birth (*n*, %)	19 (27.9%)
Previous surgery for endometriosis (*n*, %)	30 (44.1%)
Concomitant endometriomas (*n*, %)	32 (47.1%)
Use of hormonal therapies at the time of study inclusion and surgical approach (*n*, %)	50 (73.5%)
- oral estroprogestin pill	13
- contraceptive vaginal ring	2
- desogestrel	5
- norethindrone acetate	15
- dienogest	9
- etonogestrel-releasing implant	3
- levonorgestrel-releasing intrauterine device	2
- gonadotropin-releasing hormone analogue	1

**Table 2:** Diagnostic performance of 3D-RWC-TVS and CTC in the diagnosis of rectosigmoid endometriosis.

	3D-RWC-TVS	CTC
Sensitivity ^a^	70.27% (53.02% to 84.13%)	94.59% (81.81% to 99.34%)
Specificity ^a^	96.55% (82.24% to 99.91%)	93.10% (77.23% to 99.15%)
Positive likelihood ratio ^b^	20.38 (2.94 to 141.42)	13.72 (3.59 to 52.36)
Negative likelihood ratio ^b^	0.31 (0.19 to 0.51)	0.06 (90.02 to 0.22)
Positive predictive value ^a^	96.30% (78.93% to 99.45%)	94.59% (82.09% to 98.53%)
Negative predictive value ^a^	71.79% (60.69% to 80.76%)	93.10% (77.75% to 98.12%)
Accuracy ^a^	81.82% (70.39% to 90.24%)	93.94% (85.20% to 98.32%)

^a^ Values presented as percentage and 95% confidence interval; ^b^ Values presented as ratio and 95% confidence interval; 3D-RWC-TVS: Three-dimensional rectal water contrast transvaginal ultrasonography; CTC: Computed colonography.

**Table 3:** Difference between the size of the largest nodule estimated by imaging techniques and that measured on histopathology.

Location	Length on Histology (mm; Mean ±SD)	3D-RWC-TVS		CTC		*p* ^c^
		Difference (mm; mean, 95% CI) ^a^	LA ^b^	Difference (mm; Mean, 95% CI) ^a^	LA ^b^	
All (*n* = 37)	22.3 ± 8.7	−3.2 (−6.0 to −0.3)	−17.3 to 11.1	−1.0 (−2.1 to 0.1)	−6.4 to 4.5	0.099
Anterior lower rectum (*n* = 7)	22.1 ± 12.4	−1.0 (−3.3 to 1.4)	−5.5 to 3.3	−1.0 (−3.7 to 1.7)	−6.0 to 4.0	1.000
Anterior upper rectum (*n* = 13)	26.9 ± 11.3	−1.0 (−3.2 to 1.2)	−6.2 to 4.2	−1.3 (−2.7 to 0.2)	−4.4 to 1.7	0.836
Rectosigmoid junction (*n* = 4)	20.3 ± 3.1	1.6 (−3.5 to 6.8)	−2.4 to 5.7	1.0 (−1.5 to 3.5)	−3.1 to 3.6	0.728
Sigmoid (*n* = 17)	22.0 ± 6.4	−7.2 (−15.4 to −3.2)	−16.4 to 9.1	−1.4 (−3.8 to 1.1)	−8.2 to 6.3	0.060

^a^ Mean difference calculated by subtracting the size of the nodule measured by the imaging technique from the size of the nodule measured on histology; ^b^ Limits of agreement (LA) calculated as a mean difference ±2 SDs of the difference. ^c^ Comparison of the mean difference of 3D-RWC-TVS with that of CTC. 3D-RWC-TVS: Three-dimensional rectal water contrast transvaginal ultrasonography; CTC: Computed colonography

**Table 4:** Difference between the lower margin of the lowest rectosigmoid nodule and the anal verge estimated by imaging techniques and that measured on histopathology.

Location	Distance at Surgery (mm; mean ±SD)	3D-RWC-TVS		CTC		*p* ^c^
		Difference (mm; Mean, 95% CI) ^a^	LA ^b^	Difference (mm; Mean, 95% CI) ^a^	LA ^b^	
All (*n* = 37)	142.7 ± 45.3	−16.5 (−28.7 to −4.2)	−75.7 to 43.4	3.3 (−10.1 to 16.7)	−57.3 to 62.1	0.030
Anterior lower rectum (*n* = 7)	90.7 ± 10.9	3.1 (−14.1 to 20.4)	−28.7 to 32.1	6.6 (−0.1 to 13.4)	−5.9 to 19.2	0.653
Anterior upper rectum (*n* = 13)	131.3 ± 16.5	−25.6 (−58.2 to 7.1)	−34.8 to 11.0	13.8 (−30.3 to 58.1)	−91.0 to 11.2	0.125
Rectosigmoid junction (*n* = 4)	138.3 ± 7.6	−6.6 (−32.5 to 19.1)	−27.1 to 13.7	−11.7 (−30.6 to 7.3)	−27.0 to 0.4	0.667
Sigmoid (*n* = 17)	165.7 ± 47.5	−23.8 (−43.1 to −4.4)	−73.1 to 16.8	−4.5 (−13.4 to 4.5)	−26.3 to 23.0	0.048

^a^ Mean difference calculated by subtracting distance between the nodule and anal verge measured by imaging technique from distance between the nodule and anal verge measured at surgery. ^b^ Limits of agreement (LA) calculated as mean difference ±2 SDs of the difference. ^c^ Comparison of the mean difference of 3D-RWC-TVS with that of CTC. 3D-RWC-TVS: Three-dimensional rectal water contrast transvaginal ultrasonography; CTC: Computed colonography.

## Supplementary Materials

The following are available online at https://www.mdpi.com/2075-4418/10/4/252/s1, Table S1: Presence and intensity of pain and intestinal symptoms in the study population (n = 68), Table S2: Diagnostic performance of 3D-RWC-TVS and CTC in the diagnosis of multifocal rectosigmoid endometriosis.
