# Supplementary material for: A Prospective Study Comparing Three-Dimensional Rectal Water Contrast Transvaginal Ultrasonography and Computed Tomographic Colonography in the Diagnosis of Rectosigmoid Endometriosis
Source: Diagnostics (Basel). 2020 Apr 24;10(4):252. doi: 10.3390/diagnostics10040252 (PMC7236009; doi:10.3390/diagnostics10040252)
Supplement: Supplementary file 1 [file diagnostics-10-00252-s001.zip › Supplementary Table 2.docx]

|  | **3D-RWC-TVS** | **CTC** |
| --- | --- | --- |
| **Sensitivity** **^a^** | 83.33% (35.88% to 99.58%) | 100% (54.07% to 100.00%) |
| **Specificity ^a^** | 100% (94.22% to 100.00%) | 100% (94.22% to 100.00%) |
| **Positive likelihood ratio ^b^** | - | - |
| **Negative likelihood ratio ^b^** | 0.17 (0.003 to 1.00) | 0.00 |
| **Positive predictive value ^a^** | 1.0 | 1.0 |
| **Negative predictive value ^a^** | 98.41% (91.20% to 99.73%) | 100% |
| **Accuracy ^a^** | 98.53% (92.08% to 99.96%) | 100% (94.72% to 100.00%) |

^a^ Values presented as percentage and 95% confidence interval.

^b^ Values presented as ratio and 95% confidence interval.

3D-RWC-TVS: Three-dimensional rectal water contrast transvaginal ultrasonography; CTC: Computed colonography

**Supplementary Table 2.** Diagnostic performance of 3D-RWC-TVS and CTC in the diagnosis of multifocal rectosigmoid endometriosis
