# Supplementary material for: A Prospective Study Comparing Three-Dimensional Rectal Water Contrast Transvaginal Ultrasonography and Computed Tomographic Colonography in the Diagnosis of Rectosigmoid Endometriosis
Source: Diagnostics (Basel). 2020 Apr 24;10(4):252. doi: 10.3390/diagnostics10040252 (PMC7236009; doi:10.3390/diagnostics10040252)
Supplement: Supplementary file 1 [file diagnostics-10-00252-s001.zip › Supplementary Table 1.docx]

|  | **Number of patients suffering the symptom** | **Intensity of the symptom (mean ± SD)** |
| --- | --- | --- |
| **Dysmenorrhea** | 33/33 * | 6.3 ± 2.0 |
| **Deep dyspareunia** | 48/61 ** | 5.6 ± 2.4 |
| **Non-menstrual pelvic pain** | 46 | 4.2 ± 2.0 |
| **Dyschezia** | 40 | 4.2 ± 1.9 |
| **Diarrhea** | 23 | 3.9 ± 1.6 |
| **Constipation** | 26 | 4.1 ± 2.2 |
| **Abdominal bloating** | 39 | 1.9 ± 2.9 |
| **Intestinal cramping** | 24 | 3.1 ± 1.4 |
| **Passage of mucus** | 18 | 2.7 ± 1.3 |

- All the other patients were using hormonal therapies causing amenorrhea; ** 7 patients were not sexually active;

**Supplementary Table 1.** Presence and intensity of pain and intestinal symptoms in the study population (n=68)
